# Identification and Functional Analysis of *CAP* Genes from the Wheat Stripe Rust Fungus *Puccinia striiformis* f. sp. *tritici*

**DOI:** 10.3390/jof9070734

**Published:** 2023-07-07

**Authors:** Mengxin Zhao, Yanhui Zhang, Hualong Guo, Pengfei Gan, Mengmeng Cai, Zhensheng Kang, Yulin Cheng

**Affiliations:** 1 State Key Laboratory of Crop Stress Biology for Arid Areas, College of Plant Protection, Northwest A&F University, Xianyang 712100, China; 2 College of Life Sciences, Northwest A&F University, Xianyang 712100, China; 3 Key Laboratory of Plant Hormones and Development Regulation of Chongqing, School of Life Sciences, Chongqing University, Chongqing 401331, China

**Keywords:** wheat rust, virulence factor, CAP family, secreted protein

## Abstract

Cysteine-rich secretory proteins (C), antigen 5 (A), and pathogenesis-related 1 proteins (P) comprise widespread CAP superfamily proteins, which have been proven to be novel virulence factors of mammalian pathogenic fungi and some plant pathogens. Despite this, the identification and function of CAP proteins in more species of plant pathogens still need to be studied. This work presents the identification and functional analysis of CAP superfamily proteins from *Puccinia striiformis* f. sp. *tritici* (*Pst*), an important fungal pathogen that causes wheat stripe rust on wheat worldwide. A total of six *CAP* genes were identified in the *Pst* genome, designated as *PsCAP1*–*PsCAP6*. Five PsCAP proteins, including PsCAP1, PsCAP2, PsCAP3, PsCAP4, and PsCAP5, have N-terminal signal peptides secreted with the yeast signal sequence trap assay. Single-nucleotide polymorphism (SNP) analysis indicated that they showed a low level of intraspecies polymorphism. The expression abundance of *PsCAP* genes at different *Pst* infection stages was detected by RT-qPCR, and most of them were highly expressed during *Pst* infection on wheat and also *Pst* sexual reproduction on barberry (*Berberis shensiana*). Noticeably, the silencing of these six *PsCAP* genes by BSMV-mediated HIGS indicated that *PsCAP1*, *PsCAP4*, and *PsCAP5* contribute significantly to *Pst* infection in wheat. These results indicate that PsCAP proteins may act as virulence factors during *Pst* infection, which also provides insights into *Pst* pathogenicity.

## 1. Introduction

The CAP protein superfamily is derived from proteins with great sequence similarity, including cysteine-rich secretory protein (CRISP) in vertebrates, antigen 5 (Ag5) in insects, and pathogenicity-related protein 1 (PR-1) in plants [1,2]. All members of the CAP protein superfamily contain a highly conserved CAP domain that exhibits a unique alpha–beta–alpha sandwich fold [2]. The biochemical activity and action mechanism of the CAP domain remained a mystery until the finding in the budding yeast *Saccharomyces cerevisiae* [3]. *S. cerevisiae* has three CAP proteins designated as PRY1–3, and biochemical analysis showed that they can bind sterols and assist in protein export [3]. In addition, expression of the mammalian CRISP2 protein can restore the defects of PRY mutants, indicating that the conserved CAP domain is necessary and sufficient for sterol binding and lipid export [3]. Moreover, many CAP proteins have been proven to have N-terminal signaling peptides and can be secreted outside of the cell [1], while some of them are deficient in secretion function [1], which suggests that CAP proteins may have functional diversity due to different localizations. These structural components of CAP proteins form the basic ingredients for the biological functions of CAP proteins. Incorporating the signature sequences and motifs, more CAP proteins in different species can be identified using Pfam to advance the study of the identification of CAP proteins in different species.

CAP proteins are found in the majority of living organisms and are involved in multiple cellular processes, including reproduction, prostate and brain cancer in mammals, and immune defense in plants [1]. In addition, CAP proteins have also been characterized as novel virulence factors in mammalian pathogenic fungi [2]. In the ascomycete *Fusarium oxysporum*, the mutant-lacking *Fpr1* that encodes a secreted CAP protein reduced virulence in mammalian hosts [4]. The PR-1-like proteins of Rbe1 and Rbt4 from the mammalian pathogen *Candida albicans* were reported to interact with the host neutrophils contributing to pathogenesis and impeding mammalian cell clearance of the pathogen [5]. Many plant fungal pathogens display expansion of CAP proteins [2], and some *CAP* genes from *Moniliophthora perniciosa* were proven to be highly and specifically expressed during the interaction with the host cacao [6], which indicates that CAP proteins may also play important roles in the virulence of the plant fungal pathogen. The PR-1-like protein FgPR-1L-4 from wheat pathogen *Fusarium graminearum* is involved in pathogen–host interaction and affects fungal virulence in the host, which is the first example that proves non-plant PR-1 protein’s role as a pathogenicity factor to a host [7]. New research reported that CcCAP1 from *Cytospora chrysosperma* causes canker disease in poplar, mainly localized to the plant nucleus to suppress plant immunity, and the conserved CAP domain was sufficient for its function [8]. These results indicate that CAP proteins play pathogenic roles in plant-pathogenic fungi. Despite this, the identification and function of CAP proteins in more plant pathogenic fungi still need to be studied.

Rust fungi (*Uredinales* or *Pucciniales*) comprise the largest group of plant pathogenic fungi and mainly infect wheat, oat, barley, and other cereal crops and weeds [9]. Wheat stripe rust is caused by *Puccinia striiformis* f. sp. *tritici* (*Pst*), which is one of the main yield-limiting factors affecting wheat production globally [9,10]. The wheat stripe rust can cause 10–70% of wheat yield loss, which mainly depends on epidemiology, environmental climate, wheat cultivars, and race of pathogens [9,11,12]. The evolution and migration of newly generated *Pst* virulent races are occurring at an increasing rate. These leads to the loss of disease resistance in multiple wheat varieties. Thus, a better understanding of the molecular mechanism used by *Pst* pathogenicity is important for the development of more effective and durable control of wheat stripe rust. *Pst* is an obligate biotrophic parasite that is entirely dependent on the host plant to complete its growth and reproduction. The main stage of the life cycle of *Pst* is the asexual reproduction stage, that is, from the inoculation of urediniospores in wheat plants to the production of new urediniospores [12,13]. This asexual stage is usually divided into three stages: penetration stage, biotrophic/parasitic stage, and sporulation stage [14]. In the parasitic stage, it is very important to establish the elaborate relationships between *Pst* and wheat, and some of the up-regulated genes have been proven to be virulence factors for *Pst* infection at this stage [15,16,17].

Given the important role of CAP proteins in the virulence of plant fungal pathogens, this study identified six genes encoding putative CAP protein in the *Pst* genome, designated as *PsCAP1*–*PsCAP6*, which have the conserved CAP domains and belong to the PR-1-like subfamily in plants. Sequences and structural features of these genes and their expression profile throughout *Pst* development were analyzed. The functions of *PsCAP* genes were investigated by gene silencing using BSMV-mediated HIGS. We revealed that CAP proteins are novel virulence factors during the *Pst* infection of wheat plants.

## 2. Materials and Methods

### 2.1. Bioinformatics Analysis of Pst CAP Genes

The sequences of *Pst CAP* genes were obtained by comparing the amino acid sequences of three CAP proteins (PRY1–3) from the model fungus *S. cerevisiae* with the NCBI database. CAP proteins in different species were searched in the Broad Institute Web Server and NCBI website by Blastp searches. The signal peptides of CAP proteins were predicted using SignalP 5.0, an online signal peptide prediction website. CLUSTALW was used to conduct multiple sequence alignment among *Pst* CAP proteins and CAP proteins in other species, and the results were viewed using JALVIEW 2.8 [18]. MEGA5 was used for creating phylogenetic trees [19], and the sequences displayed in the phylogenetic tree were downloaded from the NCBI database.

### 2.2. Sequence Polymorphism Analysis of Pst CAP Proteins

The local blast was used to identify sequences of the coding regions of each *PsCAP* gene among seven *Pst* isolates, including CYR32, PST-21, PST-43, PST-78, PST-130, PST-08/21, and PST-87/7. The genomes of the seven isolates were all downloaded from the NCBI database. DNAMAN6.0 was used to conduct multiple sequence alignments, which were then manually adjusted to minimize the number of implied mutations. MEGA5 was used to calculate the nonsynonymous substitutions (dN) and synonymous substitutions (dS), respectively, and then the ratio of dN/dS was obtained manually.

### 2.3. Strains and Plants, Gene Cloning, and Plasmid Construction

The stripe rust isolate used in this study was CYR32, and the wheat cultivar used was *Suwon11*, which is highly susceptible to CYR32 (CYR32 and *Suwon11* form a compatible interaction). Wheat was cultured at 16 °C with a standard light–dark cycle of 16 h of light (60 μmol m^−2^·s^−1^). Yeast isolate YTK12 used for secretion validation was cultured in the YPDA liquid medium at 30 °C. BSMV isolate ND-18 was used for gene silencing [20]. The pSUC2T7M13ORI (hereinafter referred to as pSUC2) vector was used to verify the yeast secretion system [21]. Specific segments of *PsCAP* genes were inserted into the γ vector for the silencing system, respectively. The CYR32-infected *Suwon11* cDNA samples were used as templates to amplify *PsCAP* genes by PCR. Primer 5.0 and online NCBI Primer-BLAST were used to design specific primers listed in Supplementary Materials Table S2.

### 2.4. Yeast Signal Peptide Secretion Validation

To identify the secretion feature of *Pst* CAP proteins, a Yeast Signal Peptide Screen Trap (YSST) assay was carried out as previously described [21,22]. Each of the N-terminal signal peptide sequences of four PsCAP proteins (PsCAP1, PsCAP2, PsCAP4, and PsCAP5) and the first 30 amino acids of the other two PsCAP proteins (PsCAP3 and PsCAP6) were fused to the vector pSUC2T7M13ORI (hereinafter referred to as pSUC2). The pSUC2-derived plasmids were transformed into yeast strain YTK12, which is defective for tryptophan biosynthesis using the lithium acetate method [23]. The pSUC2 vector, which carries the sucrose invertase gene, lacks the signal peptide, and the sucrose invertase gene can only be switched on to convert exogenous sucrose into glucose when the signal peptide is inserted. A CMD-W medium with sucrose in place of glucose was used for all transformants. To assay the secretion of invertase, positive colonies were cultured on the YPRAA medium with raffinose as the carbohydrate source. The pSUC2-Avr1b (a secreted signal peptide of Avr1b from *Phytophthora sojae*) and pSUC2-Mg87 (25bp in the N-terminal of *Magnaporthe oryzae* protein Mg87) were used as positive and negative controls, separately [24].

### 2.5. RNA Isolation and RT-qPCR Analysis

Wheat cultivar *Suwon11*, which grew to the two-leaf stage, was used to inoculate CYR32 as previously described [25]. Samples were collected at 12, 24, 48, 72, 120, 168, and 216 hpi (hours post inoculation), respectively. Urediniospores were incubated for 10 h in sterile distilled water at 9 °C for harvesting germinated urediniospores [26]. Infected barberry (*Berberis shensiana*) leaves were sampled at 11 dpi [27]. Total cellular RNA was extracted using the Quick RNA Isolation Kit (Huayueyang Biotech Co., Ltd., Beijing, China), and the operation method was referred to the product manual protocol. Then the first-strand cDNA was synthesized using an RT-PCR system (Promega, Madison, WI, USA). RT-qPCR was performed to measure the gene transcription level. The housekeeping gene of *PsEF1* (Elongation factor 1) was used as an endogenous control. Reactions were performed on a Bio-Rad CFX Manager (version 3.1). The comparative 2^−ΔΔCT^ method was used to quantify relative gene expression [28].

### 2.6. BSMV-Mediated Gene Silencing of PsCAP Genes

Barley stripe mosaic virus (BSMV)-mediated gene silencing was conducted to identify the virulence of *PsCAP* genes [20]. Capped in vitro transcripts were prepared with linearized plasmids containing the three-part genome of BSMV (α, β, γ, or recombinant γ gene) using the RiboMAX™ Large Scale RNA Production Systems-T7 (Promega, Madison, WI, USA). BSMV:α, BSMV:β, and BSMV:γ or recombinant γ vectors were mixed with 1× Fes buffer at the ratio of 1:1:1 to inoculate the two-leaf wheat seedlings, which then were cultured at 25 °C until the leaves showed the typical chlorotic mosaic symptom [20]. The phenotype was observed and photographed. BSMV:TaPDS (TaPDS, the wheat phytoene desaturase) was used as an index for BSMV infection, and the wheat seedlings inoculated with a 1× Fes buffer were used as a negative control (MOCK). The fourth leaves were further inoculated with the *Pst* isolate of CYR32 and then maintained at 16 °C. The *Pst*-infected leaves should be sampled at 24 and 120 hpi for RNA isolation, and RT-qPCR was performed to evaluate the silencing efficiency; this method was described previously. *Pst* infection phenotypes (urediospore sporulation) were recorded and photographed at 14 dpi.

## 3. Results

### 3.1. Pst Contains Six CAP Genes That Are Specifically Expanded in Rust Fungi

**Figure 1:**
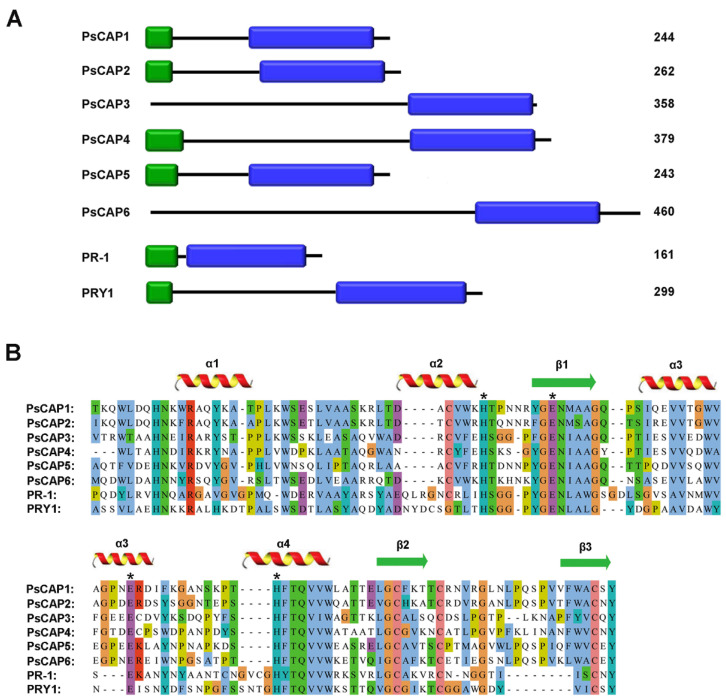
Domain architectures and sequences alignment of *Pst* CAP proteins. (**A**) Domain architectures of PsCAP1–PsCAP6. Signal peptides are represented by green blocks, and CAP domains are represented by blue blocks. The number on the right represents the size per PsCAP protein. (**B**) Sequences alignment of six CAP proteins and other known CAP proteins. The different colors of the amino acids are taken from Clustal X Colour Scheme in Jalview. The conserved secondary structural elements of CAP proteins are labeled above the sequences. The red helix represents the alpha-helix, and the green arrow represents the beta-strand. Asterisks represent the conserved residues of the CAP protein tetrad and the coordinate metals in the CAP domain [29]. PR-1: *Fusarium oxysporum* PR-1 protein (GenBank: ACV31371.1); PRY-1: *Saccharomyces cerevisiae* PRY-1 protein (GenBank: NP_012456.1).

From a BLAST search using the three CAP proteins (PRY1-3) from the model fungus *S. cerevisiae*, the queries revealed six genes encoding putative CAP proteins (designated *PsCAP1*–*PsCAP6*) in the *Pst* genome (Supplemental Table S1). The six genes were heterogeneous in size and gene structure (Supplemental Table S1), but all predicted proteins contained a C-terminal CAP domain of about 120 amino acids in size (Figure 1A and Supplemental Table S1) and shared significant sequence similarity over the CAP domain (Figure 1B). In addition to the CAP domain, four *Pst* CAP proteins, including PsCAP1, PsCAP2, PsCAP4, and PsCAP5, also contained a putative N-terminal signal peptide (Figure 1A and Supplemental Table S1).

**Figure 2:**
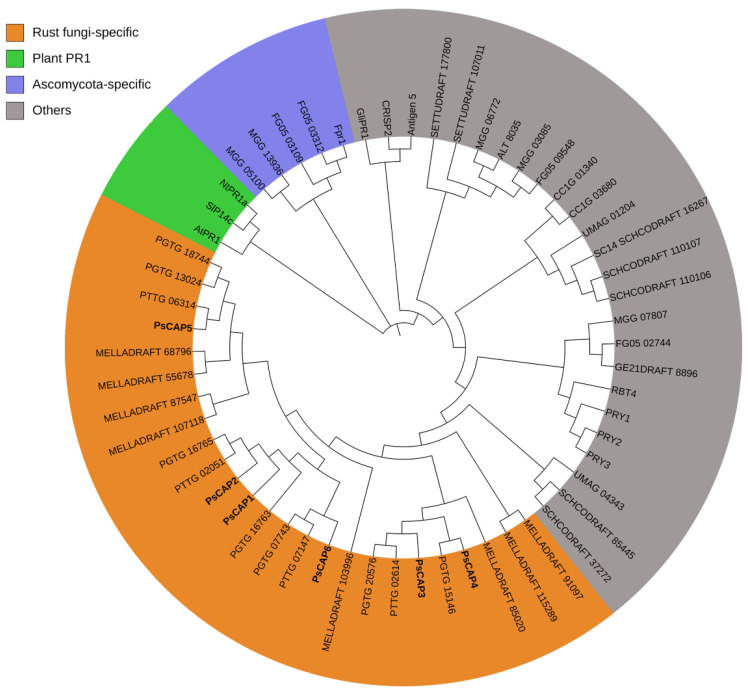
Phylogenetic tree of *Pst* CAP proteins and CAP proteins from other organisms. The phylogenetic tree was constructed based on the neighbor-joining method. The amino acid sequences displayed in the phylogenetic tree were downloaded from the NCBI database. The scale in the figure represents the evolutionary distance. The species origin of genes and GenBank accession numbers are shown as follows. Fpr1, *F. oxysporum* (GenBank: ACV31371.1); RBT4, *Candida albicans* (GenBank: AAG09789); PRY1, *S. cerevisiae* (GenBank: NP_012456.1); PRY2, *S. cerevisiae* (GenBank: NP_012938.3); PRY3, *S. cerevisiae* (GenBank: NP_012457.1); SlP14c, *Solanum lycopersicum* (GenBank: NP_001234358.1); NtPR-1a, *Nicotiana tabacum* (GenBank: BAA14220); AtPR1, *Arabidopsis thaliana* (GenBank: NP_179068.1); GliPR-1, human glioma PR-1 protein (GenBank P48060); CRISP2, *Homo sapiens* (GenBank: AAI07708.1); Tex31, *Conus textile* (GenBank: CAD36507); Antigen 5, *Dolichovespula maculate* (GenBank: AAA28301.1); FG05, *Fusarium graminearum* (GenBank: GCA_000599445); GE21DRAFT, *Neurospora crassa* (GenBank: GCA_000786625); MGG, *Magnaporthe oryzae*; UMAG, *Ustilago maydis*; SETTUDRAFT, *Setosphaeria turcica* Et28A; ALT, *Aspergillus lentulus*; SCHCODRAFT, *Schizophyllum commune* H4-8; CC1G, *Coprinopsis cinerea okayama*7#130; MELLADRAFT, *Melampsora larici-populina*.

To check the phylogenetic relationship among the six CAP proteins from *Pst* and CAP proteins from other organisms, we performed a phylogenetic analysis according to sequence alignment. We included the plant PR-1 proteins P14c from tomato [30], PR1a from tobacco [31], and AtPR-1 from *Arabidopsis* [32] in the analysis. Phylogenetic analysis indicated that all PsCAP proteins belong to a clade of rust fungi-specific CAP proteins (Figure 2). In addition, we found a clade of *ascomycota*-specific CAP proteins (Figure 2).

### 3.2. Intraspecific Variation of PsCAP Genes

To identify the intra-species polymorphism of the six *PsCAP* genes, we compared their coding regions in seven different *Pst* isolates, and the results are shown in Table 1. A total of 3–12 nucleotide substitutions but only 1–3 nonsynonymous nucleotide substitutions were observed in the six *CAP* genes (Table 1). Additionally, *PsCAP4* contains 15 nucleotide insertions/deletions among different *Pst* isolates (Supplemental Figure S1). We also calculated the ratio of nonsynonymous (dN) to synonymous (dS) substitution [33] of the six *CAP* genes. None of the six *PsCAP* genes had a ratio of dN/dS significantly greater than 1.0 (Table 1), suggesting that all these six genes are under negative selection. These results indicated that each CAP family member shows a low level of polymorphism among different *Pst* isolates.

### 3.3. Secretion Validation of Predicted Signal Peptides of CAP Proteins

**Figure 3:**
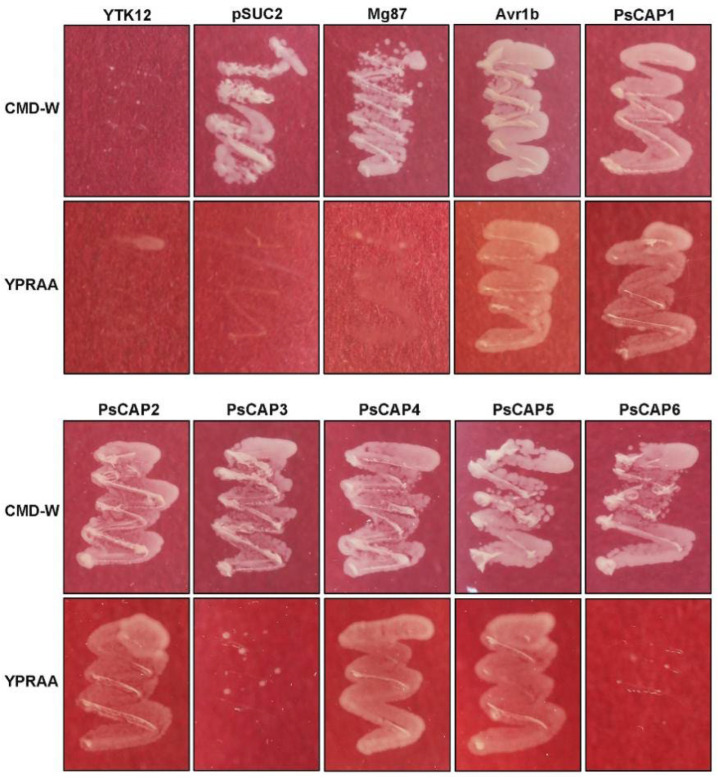
Secretion clarification of *Pst* CAP proteins. The predicted signal peptide sequences of PsCAP1, PsCAP2, PsCAP4, and PsCAP5, as well as the first 30 amino acids of PsCAP3 and PsCAP6, were fused to the pSUC2T7M13ORI vector (hereinafter referred to as pSUC2), and then the constructed vectors were transformed into yeast strain YTK12, respectively. The strains that successfully transformed with specified signal peptides:pSUC2 fusion vectors could grow on both a CMD-W medium and YPRAA medium because the signal peptides allow invertase to be secreted out of the yeast cell. Avr1b, the secreted protein from *Phytophthora sojae*, was the positive control. Mg87, a non-secreted Mg87 protein from *Magnaporthe oryzae*, was the negative control.

Many CAP proteins from other organisms have been clarified to have secreted signaling peptides [2]. We performed a yeast signal peptide screening trap [21,22] to validate the putative secretion function of the six PsCAP proteins. As shown in Figure 3, all YTK12 strains carrying pSUC2 or pSUC2-recombinant plastids can grow on a sucrose-contained CMD-W medium, indicating that the vectors of the yeast strain were successfully transformed. YTK12 strains carrying PsCAP1, PsCAP2, PsCAP4, and PsCAP5 could grow on the YPRAA medium (with raffinose as the only carbohydrate source, which requires secreted sucrose invertase for yeast growth). By contrast, PsCAP3 and PsCAP6, which have no N-terminal signal peptides, could not enable YTK12 to grow on the YPRAA medium (Figure 3). The pSUC2-Avr1b construct (Avr1b, a secreted protein from *Phytophthora sojae*) and pSUC2-Mg87 (25bp in the N-terminal of *Magnaporthe oryzae* protein Mg87) [24] were used as positive and negative controls separately (Figure 3). The results indicated that the signal peptides of PsCAP1, PsCAP2, PsCAP4, and PsCAP5 are active in the yeast-secretion system.

### 3.4. PsCAP Genes Are Highly Expressed in Pst Parasitic Stages

**Figure 4:**
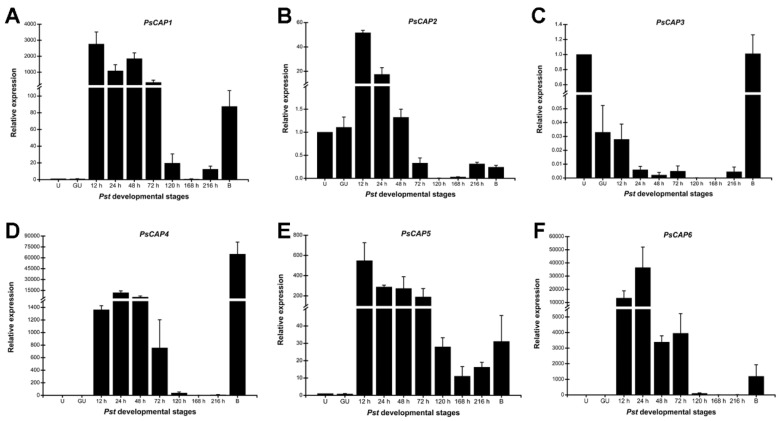
Transcription levels of *PsCAP1-PsCAP6* (**A**–**F**) during *Pst* infection stages. Wheat seedlings inoculated with CYR32 were sampled, and different stages of the relative transcript levels of *CAP* genes were calculated relative to that of the urediniospores by the comparative threshold (2^−ΔΔCT^) method. The data were normalized to the reference gene of *PsEF1*. Data are from three independent experiments ± S. E. U: urediniospores; GU: in vitro germinated urediniospores; B, infected *Berberis shensiana* (the alternate host of *Pst*).

Several CAP proteins from mammals exhibit significant expression abundance in immune-related cells and tissues [34]. In plants, PR-1 family proteins are strikingly up-regulated during pathogens infection [35]. RT-qPCR was conducted to verify the expression profile of *PsCAP* genes in different infection stages during the *Pst* life cycle. A specific expression pattern was shown by each *PsCAP* gene during the interaction of *Pst* CYR32 with wheat cultivar *Suwon11*. The transcript levels of four *PsCAP* genes (*PsCAP1*, *PsCAP4*, *PsCAP5*, and *PsCAP6*) proliferated during the period of 12–72 hpi, the key biotrophic stage for *Pst*–wheat interaction (Figure 4A,D–F). *PsCAP2* was up-regulated in 12 and 24 hpi (Figure 4B). In addition, we detected the expression levels of *PsCAP* genes in *Pst*-infected barberry (*Berberis shensiana*), which was the alternate host of *Pst*. Noticeably, *PsCAP1*, *PsCAP4*, *PsCAP5*, and *PsCAP6* were also highly up-regulated in *Pst*-infected barberry (Figure 4). Thus, the results indicated that *CAP* genes seem to be preferentially expressed during the infection of host plants, hinting at their possible role in *Pst* virulence.

### 3.5. Three PsCAP Genes Are Required for Pst Infection on Wheat

**Figure 5:**
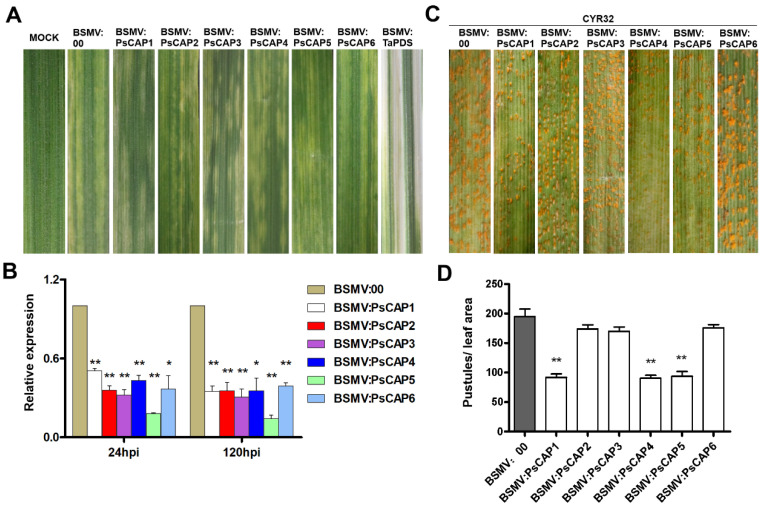
*PsCAP* genes are required for *Pst* infection. (**A**) Chlorotic mosaic symptoms on wheat seedlings inoculated with BSMV: 00 and BSMV: PsCAPs. BSMV: TaPDS (positive control)-inoculated seedlings showed severe symptoms of chlorophyll bleaching. MOCK: wheat seedlings inoculated with 1× FES buffer; (**B**) Relative transcript levels of *PsCAP*s in knockdown wheat seedlings. RNA samples were isolated from the fourth leaves of wheat seedlings pre-infected with BSMV and then inoculated with the virulent *Pst* isolate of CYR32. The data were normalized to the reference gene of *PsEF1*, with BSMV:00 standardized at 1. Data are from three independent experiments ± S. E. Differences were assessed using Student’s *t*-tests. Single/double asterisks indicate *p* < 0.05 and *p* < 0.01, respectively. (**C**) Phenotypes of the fourth leaves of virus pre-inoculated wheat seedlings infected with the isolate of CYR32. (**D**) Pustules statistics of the fourth leaves of wheat seedlings pre-inoculated with BSMV and then infected with the CYR32 isolate. Data are from three independent experiments ± S. E. Differences were assessed using Student’s *t*-tests. Double asterisks indicate *p* < 0.01.

In order to analyze whether *CAP* genes can affect the infection of *Pst* on wheat, transient silencing of these *PsCAP* genes in wheat was performed by the BSMV-induced gene-silencing technique [20,36]. The phenotypes after inoculation of recombinant BSMV on wheat seedlings are shown in Figure 5A, showing chlorotic mosaic symptoms but without significant leaf damage. Meanwhile, wheat seedlings inoculated with BSMV:TaPDS showed a severe chlorophyll bleaching phenotype, which suggested that the wheat phytoene desaturase gene *TaPDS* was successfully silenced (Figure 5A). Then, the *Pst* isolate CYR32 was inoculated on wheat seedlings pre-infected with BSMV. As shown in Figure 5C, urediospores of different degrees were generated on the leaves of the target gene-silenced wheat seedlings. The statistical results showed that BSMV:PsCAP1, BSMV:PsCAP4, and BSMV:PsCAP5 had significantly reduced pustules per unit area compared to BSMV:00 (the negative control) leaves (Figure 5D). RT-qPCR was conducted to determine the gene silencing efficiency of the six *CAP* genes, and all of them were effectively silenced (Figure 5B). These results suggested that *PsCAP1*, *PsCAP4*, and *PsCAP5* are required for *Pst* virulence by contributing to the *Pst* infection of wheat plants.

## 4. Discussion

The CAP protein superfamily is reported to be found in more than 2500 species, including most prokaryotes and eukaryotes such as bacteria, fungi, plants, and animals [2]. CAP proteins have long been reported to be involved in multiple cellular processes, including reproduction, prostate and brain cancer in mammals, and immune defense in plants [1]. In addition, there is increasing evidence that CAP proteins play an important role in the pathogenicity of both mammalian pathogenic fungi and some plant pathogens. Despite this, the identification and function of CAP proteins in more plant pathogens still need to be studied. One such plant pathogen is *Pst*, a fungus that severely threatens wheat yield worldwide. This study identified six *Pst* proteins with conserved CAP domains and homologous to plant PR-1 proteins. The characterized functions revealed that PsCAP proteins are novel virulence factors during the *Pst* infection of wheat plants.

The CAP motif is characteristic and definitive of the entire CAP superfamily and has been proven necessary and sufficient for the CAP protein’s biochemical activity and mode of action [1,3]. As shown in our results, all six PsCAP proteins contain the CAP motif with a conserved secondary structure of the alpha–beta–alpha sandwich fold [37,38]. Phylogenetic analysis in our study indicated that all PsCAP proteins belong to a clade of rust fungi-specific CAP proteins. In addition, we found there is also a clade of *ascomycota*-specific CAP proteins. These results indicate the evolutionary diversity of CAP superfamilies across species, which is consistent with the results described previously [39]. We also predicted the signal peptides among the six genes and, as shown in the results, *PsCAP1*, *PsCAP2*, *PsCAP4*, and *PsCAP5* had predicted signal peptides. The secretion function of the four predicted signal peptides was confirmed using a yeast-secretion trap assay. This study suggests that these PsCAP proteins with signal peptides are likely to be secreted by *Pst* and then may enter host plants upon *Pst* infection. Compared with CAP proteins in other species, all CRISP proteins contain a predicted signal peptide consistent with their extracellular function [1]. However, mammalian GLIPR2 proteins do not contain a predicted signal sequence, and further investigation has proven their functional sites in the intracellular Golgi membrane [40]. Considering the difference among PsCAP proteins in sequence and structure, we presumed that PsCAP proteins may have functional diversity.

In this study, RT-qPCR revealed that five *PsCAP* genes (*PsCAP1*, *PsCAP2*, *PsCAP4*, *PsCAP5*, and *PsCAP6*) were strikingly up-regulated in the early stage of *Pst* infection in wheat. The early stage of *Pst* infection is the key period for haustoria formation. During this period, *Pst* could secrete a variety of pathogenicity factors, including effectors through haustoria [17,41,42], thus affecting plant immunity and establishing a parasitic relationship between *Pst* and wheat. This strongly hints at the potential role of PsCAP proteins in the biotrophic stage of *Pst*. In addition, previous studies have reported that the majority of CAP proteins in other species showed a notable expression bias in immunity-related activities [1]. The largest number of CAP proteins (11 in total) were identified from *M. perniciosa*, a pathogen causing witches’ broom disease in cacao plants, and most of them are highly and specifically induced during infection [6]. This is consistent with the results of our study. In addition, PsCAP1, PsCAP4, PsCAP5, and PsCAP6 were also highly up-regulated in *Pst* sexual reproduction in barberry, indicating that some of the PsCAP proteins might play a role in *Pst* infection of alternate host barberry.

To investigate the role of CAP proteins in *Pst* pathogenicity, we silenced six *PsCAP* genes with the BSMV-mediated virus-silencing system separately. The results indicated that the sporulation of *PsCAP1*, *PsCAP4*, and *PsCAP5* was significantly reduced, indicating their virulence roles in *Pst* infection on wheat. Similar results were reported in CAP superfamily proteins from other species. CAP proteins have been reported as novel virulence factors in pathogenic fungi during infection of the mammalian hosts, such as RBT4 in *C. albicans* and Fpr1 in *F. oxysporum* [4,43]. Afterward, similar functions were reported from the CAP proteins of some plant pathogenic fungi. The CAP protein FgPR1L-4 from *F. graminearum*, a pathogenic fungus that causes wheat head blight, can promote fungal virulence during wheat infection [7]. Two of the three CAP proteins (VmPR1a and VmPR1c) identified in *Valsa mali* are virulence factors in the pathogenic infection course of host apples [44]. Deleting the CAP protein CcCAP1 from *C. chrysosperma*, the agent that causes poplar canker disease, decreases pathogen virulence during the infection process [8]. Importantly, the authors found that the conserved CAP domain is essential for CcCAP1′s virulence activity [8]. However, in our results, there was no significant sporulation reduction in PsCAP2-, PsCAP3-, and PsCAP6-silenced wheat seedlings, implying that they do not affect *Pst* pathogenicity. Presumably, this is due to functional redundancy as described in *C. albicans* and *C. chrysosperma* [5,8]. According to the results presented above, it seems that the secreted PsCAP proteins act as effectors to attack the host’s immune system, thereby causing susceptibility. In plant species, PR-1 proteins have been characterized as markers of induced defense against pathogens. Gamir and colleagues provided genetic and biochemical evidence for the capacity of PR-1 proteins to bind sterols and demonstrated that the inhibitory effect of plant PR-1 on pathogen growth is caused by sterol sequestration from pathogens [45]. Therefore, it is speculated that the pathogenic mechanism of secreted PsCAP proteins may be that PsCAP proteins act as fungal effectors by sequestering host plant sterols. The precise regulatory mechanism of each PsCAP family member needs further investigation.

In conclusion, this study identified six genes (*PsCAP1*–*PsCAP6*) encoding CAP subfamily proteins and homologous to plant PR-1 proteins in *Pst*. We clarified their virulence roles in the *Pst*–wheat interaction. Five of the six PsCAP proteins have N-terminal signaling peptides that may be secreted by *Pst* during infection. The expression profile indicated that most of the *PsCAP* genes are preferentially up-regulated during the infection of host plants. BSMV-mediated HIGS revealed that the sporulation of *PsCAP1*, *PsCAP4*, and *PsCAP5* was significantly reduced in the gene-silenced plants, indicating their roles in *Pst* infection. Our results suggest that CAP proteins may act as virulence factors during *Pst* infection, which also provides significant insights into *Pst* pathogenicity.

## Figures and Tables

**Table 1:** SNP analysis of *PsCAP* genes among seven *Pst* isolates.

	Single Nucleotide Polymorphisms (SNPs) ^a^			Compute Overall Mean Distance Estimation ^e^		
Gene Name	NS ^b^	NNS ^c^	Insertion /Deletin ^d^	dN	dS	dN/dS
PsCAP1	3	1	—	0.0005	0.0033	0.15
PsCAP2	6	3	—	0.0014	0.0045	0.31
PsCAP3	12	2	—	0.0007	0.0185	0.04
PsCAP4	3	1	15	0.0006	0.0029	0.21
PsCAP5	10	3	—	0.0019	0.0168	0.11
PsCAP6	9	2	—	0.0009	0.0092	0.10

^a^ SNPs were done in seven different Pst isolates including one Chinese isolate (CYR32), four US isolates (PST-21, PST-43, PST-78 and PST-130) and two UK isolates (PST-08/21 and PST-87/7); ^b^ Number of total nucleotide substitutions (NS); ^c^ Number of nonsynonymous nucleotide substitutions (NNS); ^d^ Number of nucleotide insertion/deletion; ^e^ Compute overall mean distance estimation was done by MEGA5.

## Data Availability

All data generated or analyzed in this research process are included in this published article and its Supplementary Materials.

## Supplementary Materials

The following supporting information can be downloaded at: https://www.mdpi.com/article/10.3390/jof9070734/s1, Figure S1: Sequence alignment of PsCAP4 among 7 *Pst* isolates; Table S1: Characteristics of six *PsCAP* genes identified in the *Pst* genome; Table S2: List of specific primers designed in this study.
