# Supplementary material for: Identification and Functional Analysis of CAP Genes from the Wheat Stripe Rust Fungus Puccinia striiformis f. sp. tritici
Source: J Fungi (Basel). 2023 Jul 7;9(7):734. doi: 10.3390/jof9070734 (PMC10381272; doi:10.3390/jof9070734)
Supplement: Supplementary file 1 [file jof-09-00734-s001.zip › Table S1 Characteristics of six PsCAP genes identified in the Pst genome.pdf]

Table S1. Characteristics of six *PsCAP* genes identified in the *Pst* genome.

**Gene name**	**Ensembl Fungi Locus**	**CDS (bp)**	**No. of introns**	**Protein (aa)**	**SP* (aa)**	**CAPdomain （aa）**	**GenBank ID**
PsCAP1	PSTG_01738	735	5	244	1-22	103-221	KNF05109
PsCAP2	PSTG_01739	789	5	262	1-22	121-239	KNF05110
PsCAP3	PSTG_06516	1077	6	358	—	241-356	KNF00343
PsCAP4	PSTG_11017	1140	9	379	1-31	238-355	KNE95652
PsCAP5	PSTG_13342	732	4	243	1-25	100-218	KNE93302
PsCAP6	PSTG_15884	1383	5	460	—	283-401	KNE90672

*SP, signal peptide
