# Supplementary material for: Identification and Functional Analysis of CAP Genes from the Wheat Stripe Rust Fungus Puccinia striiformis f. sp. tritici
Source: J Fungi (Basel). 2023 Jul 7;9(7):734. doi: 10.3390/jof9070734 (PMC10381272; doi:10.3390/jof9070734)
Supplement: Supplementary file 1 [file jof-09-00734-s001.zip › Table S2 List of specific primers designed in this study.pdf]

Table S2. List of specific primers designed in this study.

Primer name	Primer 5'-3'	Purpose
PsEF1-qRT-F	TTCGCCGTCCGTGATATGAGACAA	The endogenous reference for normalization in RT-qPCR
PsEF1-qRT-R	ATGCGTATCATGGTGGTGGAGTGA	The endogenous reference for normalization in RT-qPCR
PsCAP1-qRT-F	GCCATCACAAGACGGTAACAA	qRT-PCR for validation of the expression patterns of PsCAP1
PsCAP1-qRT-R	GGAAGCCGCAACCAAACT	qRT-PCR for validation of the expression patterns of PsCAP1
PsCAP1-vigs-F1	CCTTAATTAACTTCGCAGTCACGTCCCA	Clone PsCAP1 to the r vector for HIGS
PsCAP1-vigs-R	TATGCGGCCGCGAGCCCGCCACTTGTTGT	Clone PsCAP1 to the r vector for HIGS
PsCAP2-qRT-F	CCAGCCCGCTCAAATCTA	qRT-PCR for validation of the expression patterns of PsCAP2
PsCAP2-qRT-R	GGGTTGCCATTGGTAGGAC	qRT-PCR for validation of the expression patterns of PsCAP2
PsCAP2-vigs-F	CCTTAATTAAGTTGTCACAGGCTGGGTGG	Clone PsCAP2 to the r vector for HIGS
PsCAP2-vigs-R	TATGCGGCCGCCGGCAGGTGGCTTTATGG	Clone PsCAP2 to the r vector for HIGS
PsCAP3-qRT-F	GAAGGCGGTTTCTCCAGC	qRT-PCR for validation of the expression patterns of PsCAP3
PsCAP3-qRT-R	GGCGAAGCGATTCTTTGTAT	qRT-PCR for validation of the expression patterns of PsCAP3
PsCAP3-vigs-F	CCTTAATTAAAGCAGCAAATCTTCAATCCTT	Clone PsCAP3 to the r vector for HIGS
PsCAP3-vigs-R	TATGCGGCCGCACGGGCACGAGACCAGA	Clone PsCAP3 to the r vector for HIGS
PsCAP4-qRT-F	ACCGTCAATCCGAACTCTGG	qRT-PCR for validation of the expression patterns of PsCAP4
PsCAP4-qRT-R	TGGCACGATGGTGTATGTCC	qRT-PCR for validation of the expression patterns of PsCAP4
PsCAP4-vigs-F	CCTTAATTAAAAGCTGAACTCGCCGAAG	Clone PsCAP4 to the r vector for HIGS
PsCAP4-vigs-R	TATGCGGCCGCCGCAGGTTGAGAATGGTCT	Clone PsCAP4 to the r vector for HIGS
PsCAP5-qRT-F	CGACAGCCCAACGACTT	qRT-PCR for validation of the expression patterns of PsCAP5
PsCAP5-qRT-R	TTGAGGCAACCAAACACC	qRT-PCR for validation of the expression patterns of PsCAP5
PsCAP5-vigs-F	CCTTAATTAACGGACCGTCAATAGTCAAGC	Clone PsCAP5 to the r vector for HIGS
PsCAP5-vigs-R	TATGCGGCCGCGCAAGCAGCAGCAAGTCG	Clone PsCAP5 to the r vector for HIGS
PsCAP6-qRT-F	CGAATCACCACCATCACTACAT	qRT-PCR for validation of the expression patterns of PsCAP6
PsCAP6-qRT-R	GGATAAGATGGCACAGGGTT	qRT-PCR for validation of the expression patterns of PsCAP6
PsCAP6-vigs-F	CCTTAATTAAAGTCGTGCTCGCTTGGGT	Clone PsCAP6 to the r vector for HIGS
PsCAP6-vigs-R	TATGCGGCCGCCACATCCGATTTGCACAGTTT	Clone PsCAP6 to the r vector for HIGS
PsCAP1-sp-F	GGAATTCATGACCAACAAAATCATTTTCGT	Clone PsCAP1-sp to pSUC2 for secretion validation
PsCAP1-sp-R	CCGCTCGAGGGCCAAAGAGAATTTGGAAAGT	Clone PsCAP1-sp to pSUC2 for secretion validation

PsCAP2-sp-F	GGAATTCATGACCAACACGATTACTTTCGTCG	Clone PsCAP2-sp to pSUC2 for secretion validation
PsCAP2-sp-R	CCGCTCGAGGGCCAAGGAGGTTTGGGAAAG	Clone PsCAP2-sp to pSUC2 for secretion validation
PsCAP3-sp-F	GGAATTCATGGTTTGTTATTTCTCAACCTCCC	Clone PsCAP3-sp to pSUC2 for secretion validation
PsCAP3-sp-R	CCGCTCGAGGCTGCTCTGGGTTTTGGCTG	Clone PsCAP3-sp to pSUC2 for secretion validation
PsCAP4-sp-F	GGAATTCATGAGTAGCAAATCTTCAATCTTTT	Clone PsCAP4-sp to pSUC2 for secretion validation
PsCAP4-sp-R	CCGCTCGAGTGAAGATGAAGATGGGAGATGA	Clone PsCAP4-sp to pSUC2 for secretion validation
PsCAP5-sp-F	GGAATTCATGGCCGCTCTCTTTTCTGG	Clone PsCAP5-sp to pSUC2 for secretion validation
PsCAP5-sp-R	CCGCTCGAGTGCGTGTAGTAGTTGAAGAGAGAGT	Clone PsCAP5-sp to pSUC2 for secretion validation
PsCAP6-sp-F	GGAATTCATGACACATAGTTCCTCCGGTT	Clone PsCAP6-sp to pSUC2 for secretion validation
PsCAP6-sp-R	CCGCTCGAGAGAAGTTGATGTAGTGATGGTGGT	Clone PsCAP6-sp to pSUC2 for secretion validation
