# Supplementary figures and images for: Identification and Functional Analysis of CAP Genes from the Wheat Stripe Rust Fungus Puccinia striiformis f. sp. tritici
Source: J Fungi (Basel). 2023 Jul 7;9(7):734. doi: 10.3390/jof9070734 (PMC10381272; doi:10.3390/jof9070734)

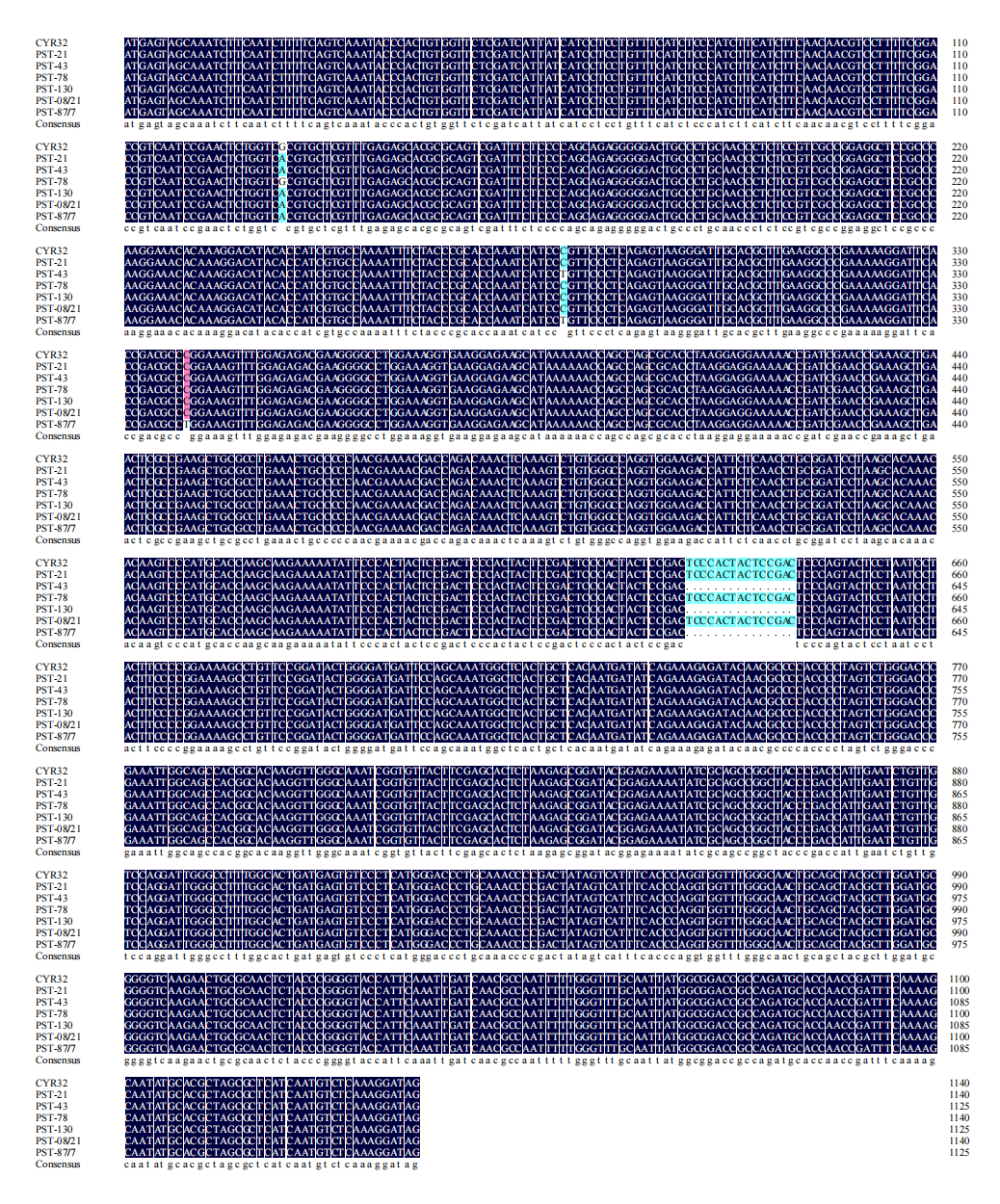

Supplement: Supplementary file 1 [file jof-09-00734-s001.zip › Figure S1 PsCAP SNP.png]
